# Supplementary material for: Comparative genomics provides new insights into the diversity, physiology, and sexuality of the only industrially exploited tremellomycete: Phaffia rhodozyma
Source: BMC Genomics. 2016 Nov 9;17:901. doi: 10.1186/s12864-016-3244-7 (PMC5103461; doi:10.1186/s12864-016-3244-7)
Supplement: Additional file 6: — List of orphan genes with links to PFAM (related to Additional file 1: Table S1). (ZIP 1428 kb) [file 12864_2016_3244_MOESM6_ESM.zip › BLAST_HTML_FTR/G04985_P.html]

BLAST Search Results


```
BLASTP 2.2.27+


Reference:
Stephen F. Altschul, Thomas L. Madden, Alejandro A. Schäffer,
Jinghui Zhang, Zheng Zhang, Webb Miller, and David J. Lipman (1997),
"Gapped BLAST and PSI-BLAST: a new generation of protein database
search programs", Nucleic Acids Res. 25:3389-3402.


Reference for
composition-based statistics:
Alejandro A. Schäffer, L. Aravind, Thomas L. Madden, Sergei
Shavirin, John L. Spouge, Yuri I. Wolf, Eugene V. Koonin, and
Stephen F. Altschul (2001), "Improving the accuracy of PSI-BLAST
protein database searches with composition-based statistics and
other refinements", Nucleic Acids Res. 29:2994-3005.


Database: nr
           71,551,133 sequences; 26,053,659,533 total letters


Query= G04985_P

Length=608
                                                                      Score     E
Sequences producing significant alignments:                          (Bits)  Value

emb|CDZ97489.1|  hypothetical protein [Xanthophyllomyces dendrorh...  1245    0.0  
emb|CED84443.1|  hypothetical protein [Xanthophyllomyces dendrorh...  57.8    3e-05
emb|CDZ96936.1|  hypothetical protein [Xanthophyllomyces dendrorh...  56.6    3e-05
emb|CED82137.1|  hypothetical protein [Xanthophyllomyces dendrorh...  53.1    6e-04
emb|CDZ96473.1|  hypothetical protein [Xanthophyllomyces dendrorh...  48.9    0.016
gb|KNC99826.1|  hypothetical protein SPPG_05200 [Spizellomyces pu...  45.8    0.12 
gb|KLT45648.1|  hypothetical protein CC85DRAFT_325375 [Trichospor...  45.8    0.14 
emb|CUA77612.1|  hypothetical protein RSOLAG22IIIB_02629 [Rhizoct...  45.4    0.19 
gb|EUC53667.1|  hypothetical protein RSOL_015180 [Rhizoctonia sol...  44.3    0.39 
gb|KEP54147.1|  hypothetical protein V565_021480 [Rhizoctonia sol...  44.3    0.39 
gb|KDQ63606.1|  hypothetical protein JAAARDRAFT_29627 [Jaapia arg...  44.3    0.41 
ref|WP_037173015.1|  hypothetical protein [Rhodococcus fascians]      42.7    0.66 
ref|XP_007861036.1|  hypothetical protein GLOTRDRAFT_135328 [Gloe...  43.5    0.78 
ref|WP_037147068.1|  hypothetical protein [Rhodococcus fascians]      41.2    2.0  
gb|KIM67191.1|  hypothetical protein SCLCIDRAFT_1210691 [Sclerode...  41.6    2.4  
emb|CDZ96692.1|  hypothetical protein [Xanthophyllomyces dendrorh...  41.2    2.8  
ref|WP_052329990.1|  hypothetical protein [Thermicanus aegyptius]     41.2    3.2  
emb|CEL57395.1|  hypothetical protein RSOLAG1IB_02134 [Rhizoctoni...  41.2    3.5  
ref|WP_005581872.1|  Group 1 glycosyl transferase [Clostridium ul...  40.8    4.2  
ref|WP_032405444.1|  hypothetical protein [Rhodococcus fascians]      39.7    6.4  


 >emb|CDZ97489.1| hypothetical protein [Xanthophyllomyces dendrorhous]
Length=607

 Score = 1245 bits (3221),  Expect = 0.0, Method: Compositional matrix adjust.
 Identities = 606/607 (99%), Positives = 606/607 (99%), Gaps = 0/607 (0%)

Query  1    MNQKGPTRESLQMLKRVSLQKLCKSLFIKGANKKSVEIIEVILAHYAQKDIEVEEAKQIA  60
            MNQKGPTRESLQMLKRVSLQKLCKSLFIKGANKKSVEIIEVILAHYAQKDIEVEEAKQIA
Sbjct  1    MNQKGPTRESLQMLKRVSLQKLCKSLFIKGANKKSVEIIEVILAHYAQKDIEVEEAKQIA  60

Query  61   PVIPSERAERVIPASRIEATKRAATIEATREPRTIVRGEGAGQAIETDVRETNITRHESS  120
            PVIPSERAERVIPASRIEATKRAATIEATREPRTIVRGEGAGQAIETDVRETNITRHESS
Sbjct  61   PVIPSERAERVIPASRIEATKRAATIEATREPRTIVRGEGAGQAIETDVRETNITRHESS  120

Query  121  GMENPRYEDLPMLSVVDEDKERLDLLQTHFDVRLAAHRRDLEERDATQWGQIEKRTAPRK  180
            GMENPRYEDLPMLSVVDEDKERLDLLQTHFDVRLAAHRRDLEERDATQWGQIEKRTAPRK
Sbjct  121  GMENPRYEDLPMLSVVDEDKERLDLLQTHFDVRLAAHRRDLEERDATQWGQIEKRTAPRK  180

Query  181  SIVCPQKKPIFEIYRDPSTPEQNFLAGPSMLGAAPIQLLTEQLSVNKNVLTVMESIMPLR  240
            SIVCPQKKPIFEIYRDPSTPEQNFLAGPSMLGAAPIQLLTEQLSVNKNVLTVMESIMPLR
Sbjct  181  SIVCPQKKPIFEIYRDPSTPEQNFLAGPSMLGAAPIQLLTEQLSVNKNVLTVMESIMPLR  240

Query  241  GNKVKIRSHPFVAASMASMSKRTRCLGSQLLAGRIRKEMNDRDQRLHPRASLSVLGKRQR  300
            GNKVKIRSHPFVAASMASMSKRTRCLGSQLLAGRIRKEMNDRDQRLHPRASLSVLGKRQR
Sbjct  241  GNKVKIRSHPFVAASMASMSKRTRCLGSQLLAGRIRKEMNDRDQRLHPRASLSVLGKRQR  300

Query  301  LNDAKSLVFRVSKRLSSLTAVELSSHIPKTLSVLGKRAREPTFSIPEYEFADEFSFPLPP  360
            LNDAKSLVFRVSKRLSSLTAVELSSHIPKTLSVLGKRAREPTFSIPEYEFADEFSFPLPP
Sbjct  301  LNDAKSLVFRVSKRLSSLTAVELSSHIPKTLSVLGKRAREPTFSIPEYEFADEFSFPLPP  360

Query  361  VLRLATPPIMDPHKDPWPSVGPLLSEPPKKSPSFSKKPKRLLVFEGCALTYIGMMKPGFV  420
            VLRLATPPIMDPHKDPWPSVGPLLSEPPKKSPSFSKKPKRLLVFEGCALTYIGMMKPGFV
Sbjct  361  VLRLATPPIMDPHKDPWPSVGPLLSEPPKKSPSFSKKPKRLLVFEGCALTYIGMMKPGFV  420

Query  421  PSLEQVESRKSLKCWVAVASGQRGAPCSWIQKHRPPFVQSEREAKDDERRRQEAICWRPK  480
            PSLEQVESRKSLKCWVAVASGQRGAPCSWIQKHRPPFVQSEREAKDDERRRQEAICWRPK
Sbjct  421  PSLEQVESRKSLKCWVAVASGQRGAPCSWIQKHRPPFVQSEREAKDDERRRQEAICWRPK  480

Query  481  SVFSDGHVEFAESYYEGCSFNNYLEMVGYYPGFPKSILSSSGSMRALSLSSSSSSSAWLS  540
            SVFSDGHVEFAESYYEGCSFNNYLEMVGYYPGFPKSILSSSGSMRALSLSSSSSSSAWLS
Sbjct  481  SVFSDGHVEFAESYYEGCSFNNYLEMVGYYPGFPKSILSSSGSMRALSLSSSSSSSAWLS  540

Query  541  FLPASNHNTSLFTPNSHDPKNFPKSITHYLGDIIVTKVPCKKPGWFKRVPRDPGADGPIR  600
            FLPASNHNTSLFTPNSHDPKNFPKSITHYLGDIIVTKVPCKK GWFKRVPRDPGADGPIR
Sbjct  541  FLPASNHNTSLFTPNSHDPKNFPKSITHYLGDIIVTKVPCKKSGWFKRVPRDPGADGPIR  600

Query  601  GRVAWDA  607
            GRVAWDA
Sbjct  601  GRVAWDA  607


>emb|CED84443.1| hypothetical protein [Xanthophyllomyces dendrorhous]
Length=674

 Score = 57.8 bits (138),  Expect = 3e-05, Method: Compositional matrix adjust.
 Identities = 28/57 (49%), Positives = 35/57 (61%), Gaps = 0/57 (0%)

Query  6   PTRESLQMLKRVSLQKLCKSLFIKGANKKSVEIIEVILAHYAQKDIEVEEAKQIAPV  62
           PT   L  LKRV +QKLCK+L IKGAN+K+  +I +IL HYA       E +Q  P 
Sbjct  3   PTSIELHGLKRVDVQKLCKTLGIKGANQKTEALIGLILQHYANSQSTTSEGEQAPPA  59


>emb|CDZ96936.1| hypothetical protein [Xanthophyllomyces dendrorhous]
Length=356

 Score = 56.6 bits (135),  Expect = 3e-05, Method: Compositional matrix adjust.
 Identities = 28/57 (49%), Positives = 35/57 (61%), Gaps = 0/57 (0%)

Query  6   PTRESLQMLKRVSLQKLCKSLFIKGANKKSVEIIEVILAHYAQKDIEVEEAKQIAPV  62
           PT   L  LKRV +QKLCK+L IKGAN+K+  +I +IL HYA       E +Q  P 
Sbjct  3   PTSIELHGLKRVDVQKLCKTLGIKGANQKTEALIGLILQHYANSQSTTSEGEQAPPA  59


>emb|CED82137.1| hypothetical protein [Xanthophyllomyces dendrorhous]
Length=429

 Score = 53.1 bits (126),  Expect = 6e-04, Method: Compositional matrix adjust.
 Identities = 32/75 (43%), Positives = 45/75 (60%), Gaps = 2/75 (3%)

Query  6   PTRESLQMLKRVSLQKLCKSLFIKGANKKSVEIIEVILAHYAQKDIEVEEAK-QIAPVIP  64
           PT   L  LKR  +QKLCK+L IKGAN+K+  +I+ IL HY   +  V  +  Q +  +P
Sbjct  3   PTSTELHKLKRADVQKLCKTLGIKGANQKTKNLIDRILQHYTDVEPSVSTSGLQSSASLP  62

Query  65  SERAERVIPASRIEA  79
           SE    +  AS+I+A
Sbjct  63  SEE-NSLSNASQIDA  76


>emb|CDZ96473.1| hypothetical protein [Xanthophyllomyces dendrorhous]
Length=660

 Score = 48.9 bits (115),  Expect = 0.016, Method: Compositional matrix adjust.
 Identities = 22/40 (55%), Positives = 28/40 (70%), Gaps = 0/40 (0%)

Query  7   TRESLQMLKRVSLQKLCKSLFIKGANKKSVEIIEVILAHY  46
           T   L  LKR  +QKLCK+L IKGAN+K+  +I +IL HY
Sbjct  4   TSNELNSLKRTDVQKLCKTLGIKGANQKTDTLIGLILHHY  43


>gb|KNC99826.1| hypothetical protein SPPG_05200 [Spizellomyces punctatus DAOM 
BR117]
Length=584

 Score = 45.8 bits (107),  Expect = 0.12, Method: Compositional matrix adjust.
 Identities = 22/39 (56%), Positives = 27/39 (69%), Gaps = 0/39 (0%)

Query  7   TRESLQMLKRVSLQKLCKSLFIKGANKKSVEIIEVILAH  45
           T ESL  +KR  LQ + KSL +KGANKKS E+  +IL H
Sbjct  17  TPESLASMKRADLQGMLKSLGLKGANKKSSEMASIILEH  55


>gb|KLT45648.1| hypothetical protein CC85DRAFT_325375 [Trichosporon oleaginosus]
Length=580

 Score = 45.8 bits (107),  Expect = 0.14, Method: Compositional matrix adjust.
 Identities = 21/40 (53%), Positives = 28/40 (70%), Gaps = 1/40 (3%)

Query  7   TRESLQMLKRVSLQKLCKSLFIKGANKKSVEIIEVILAHY  46
           TR+ LQ L+R  LQ+LCK+  IKGAN KS +++   LA Y
Sbjct  8   TRDELQGLRRADLQRLCKTYNIKGANAKS-DVLITTLADY  46


>emb|CUA77612.1| hypothetical protein RSOLAG22IIIB_02629 [Rhizoctonia solani]
Length=687

 Score = 45.4 bits (106),  Expect = 0.19, Method: Compositional matrix adjust.
 Identities = 23/41 (56%), Positives = 28/41 (68%), Gaps = 1/41 (2%)

Query  6   PTRESLQMLKRVSLQKLCKSLFIKGANKKSVEIIEVILAHY  46
           PTR  L+ LKR +LQ  CK L IK AN KS  +IE++L HY
Sbjct  4   PTRAQLEGLKRSALQAKCKELGIK-ANSKSEVLIELVLEHY  43


>gb|EUC53667.1| hypothetical protein RSOL_015180 [Rhizoctonia solani AG-3 Rhs1AP]
Length=630

 Score = 44.3 bits (103),  Expect = 0.39, Method: Compositional matrix adjust.
 Identities = 23/41 (56%), Positives = 28/41 (68%), Gaps = 1/41 (2%)

Query  6   PTRESLQMLKRVSLQKLCKSLFIKGANKKSVEIIEVILAHY  46
           PTR  L+ LKR +LQ  CK L IK AN KS  +I++IL HY
Sbjct  4   PTRVELEGLKRAALQSKCKELGIK-ANSKSEMLIDLILEHY  43


>gb|KEP54147.1| hypothetical protein V565_021480 [Rhizoctonia solani 123E]
Length=632

 Score = 44.3 bits (103),  Expect = 0.39, Method: Compositional matrix adjust.
 Identities = 23/41 (56%), Positives = 28/41 (68%), Gaps = 1/41 (2%)

Query  6   PTRESLQMLKRVSLQKLCKSLFIKGANKKSVEIIEVILAHY  46
           PTR  L+ LKR +LQ  CK L IK AN KS  +I++IL HY
Sbjct  4   PTRVELEGLKRAALQSKCKELGIK-ANSKSEMLIDLILEHY  43


>gb|KDQ63606.1| hypothetical protein JAAARDRAFT_29627 [Jaapia argillacea MUCL 
33604]
Length=676

 Score = 44.3 bits (103),  Expect = 0.41, Method: Compositional matrix adjust.
 Identities = 20/38 (53%), Positives = 29/38 (76%), Gaps = 1/38 (3%)

Query  6   PTRESLQMLKRVSLQKLCKSLFIKGANKKSVEIIEVIL  43
           PTRE+L+ +KR+ LQ+LCK   +K AN KS  +I++IL
Sbjct  5   PTRETLESMKRIDLQRLCKDYGVK-ANLKSEALIDLIL  41


>ref|WP_037173015.1| hypothetical protein [Rhodococcus fascians]
Length=251

 Score = 42.7 bits (99),  Expect = 0.66, Method: Compositional matrix adjust.
 Identities = 53/207 (26%), Positives = 85/207 (41%), Gaps = 42/207 (20%)

Query  193  IYRDPSTPEQNFLAGPSMLGAAPIQLLTEQLSVNKNVLTVMESIMPLRGNKVKIRSHPFV  252
            + R P   E N L G +  G A +Q +   ++ +  VLTV               S P +
Sbjct  59   LRRRPDLAESNLLCG-NAFGGAVVQAMLPAVAPDAAVLTV---------------SAPTI  102

Query  253  AASMASMSKRTRCLGSQLLAGRIRKEMNDRD--QRLHPRASLSVLGKRQRLNDAKSLVFR  310
            A   AS+++R   +       RI   +   D   R H   S SV G   R++D      R
Sbjct  103  AD--ASLAQRLGEVAELARHRRIDDALRTLDFYVRSHSVRSPSVDGAATRIDDIDDACRR  160

Query  311  VSKRLSSLTAVEL----SSHIPKTLSVLGKRAR---EPTFSIPEYEFADEFSFPLPPVLR  363
            +   +S LT+++L    + H    L+++G+ +R     T  +PE+               
Sbjct  161  IEMGMSMLTSIDLEPVVADHRGPMLAIVGQESRLVTTATVRVPEHGV-------------  207

Query  364  LATPP--IMDPHKDPWPSVGPLLSEPP  388
            +A  P   M PH+D WP+V   L+  P
Sbjct  208  IADIPRSGMRPHRDNWPAVARALAAHP  234


>ref|XP_007861036.1| hypothetical protein GLOTRDRAFT_135328 [Gloeophyllum trabeum 
ATCC 11539]
 gb|EPQ60685.1| hypothetical protein GLOTRDRAFT_135328 [Gloeophyllum trabeum 
ATCC 11539]
Length=611

 Score = 43.5 bits (101),  Expect = 0.78, Method: Compositional matrix adjust.
 Identities = 21/38 (55%), Positives = 29/38 (76%), Gaps = 1/38 (3%)

Query  6   PTRESLQMLKRVSLQKLCKSLFIKGANKKSVEIIEVIL  43
           PTRE+L+ + R  LQKLCK+L +K AN KS  +I++IL
Sbjct  5   PTREALESMSRQDLQKLCKNLGLK-ANLKSDALIDLIL  41


>ref|WP_037147068.1| hypothetical protein [Rhodococcus fascians]
Length=251

 Score = 41.2 bits (95),  Expect = 2.0, Method: Compositional matrix adjust.
 Identities = 52/207 (25%), Positives = 84/207 (41%), Gaps = 42/207 (20%)

Query  193  IYRDPSTPEQNFLAGPSMLGAAPIQLLTEQLSVNKNVLTVMESIMPLRGNKVKIRSHPFV  252
            + R P   E N   G +  G A +Q +   +  +  VLTV               S P +
Sbjct  59   LRRRPELSESNLFCG-NAFGGAVVQAMLPAVGPDAAVLTV---------------SAPTI  102

Query  253  AASMASMSKRTRCLGSQLLAGRIRKEMNDRD--QRLHPRASLSVLGKRQRLNDAKSLVFR  310
            A   AS+++R   +       RI   +   D   R H   S SV G   R++D  +   R
Sbjct  103  AD--ASLAQRLGEVAELARHRRIDDALRTLDFYVRSHSVRSPSVEGAATRIDDIDNACRR  160

Query  311  VSKRLSSLTAVELSS----HIPKTLSVLGKRAR---EPTFSIPEYEFADEFSFPLPPVLR  363
            +   +S LT+++L +    H    L+++G+ +R     T  +PE+               
Sbjct  161  IEMGMSMLTSIDLDTVVADHRGPMLAIVGQESRLVTTATVRVPEHGV-------------  207

Query  364  LATPP--IMDPHKDPWPSVGPLLSEPP  388
            +A  P   M PH+D WP+V   L+  P
Sbjct  208  IADIPRSGMRPHRDNWPAVARALAAHP  234


>gb|KIM67191.1| hypothetical protein SCLCIDRAFT_1210691 [Scleroderma citrinum 
Foug A]
Length=576

 Score = 41.6 bits (96),  Expect = 2.4, Method: Compositional matrix adjust.
 Identities = 28/81 (35%), Positives = 44/81 (54%), Gaps = 9/81 (11%)

Query  6   PTRESLQMLKRVSLQKLCKSLFIKGANKKSVEIIEVILAHYAQKDIEVEEAKQIAPVIPS  65
           P RE+L+ +KRV +QKLCK   +K AN K+  +I+++L      D       +  P  PS
Sbjct  5   PGREALEAMKRVDIQKLCKDYGVK-ANLKTEALIDLLL------DASRPPPPRSNPTQPS  57

Query  66  ERAERVIPASRIEATKRAATI  86
           E +   IP  R   ++R++ I
Sbjct  58  EHSTPGIP--RYSGSRRSSVI  76


>emb|CDZ96692.1| hypothetical protein [Xanthophyllomyces dendrorhous]
Length=441

 Score = 41.2 bits (95),  Expect = 2.8, Method: Compositional matrix adjust.
 Identities = 29/94 (31%), Positives = 49/94 (52%), Gaps = 6/94 (6%)

Query  2    NQKGPTRESLQMLKRVSLQKLCKSLFIKGANKKSVEIIEVILAHYAQKDIEVEEAKQIAP  61
            N+  P   SL+M K   LQ+L   +++  A  +S++  EV+L   A++D   +EAK+   
Sbjct  46   NRTAPLTRSLRMEKGHPLQELFMIMYLGKARSESIQQHEVVLGQCAREDRRTDEAKR---  102

Query  62   VIPSERAERVIPASRIEATKRAATIEATREPRTI  95
               +E   R   A R+  TK A  +E++R  R +
Sbjct  103  ---AEHVGRTKLAGRVGRTKLAERMESSRPNRKL  133


>ref|WP_052329990.1| hypothetical protein [Thermicanus aegyptius]
Length=381

 Score = 41.2 bits (95),  Expect = 3.2, Method: Compositional matrix adjust.
 Identities = 37/142 (26%), Positives = 63/142 (44%), Gaps = 18/142 (13%)

Query  3    QKGPTRESLQMLKRVSLQKLCKSLFIKGANKKSVEIIEVILAHYAQKDIEVEEAKQIAPV  62
            QKG     LQ +KR+ ++KL   L I G NK  +      ++H  ++ + ++++ +  P 
Sbjct  208  QKG-VHLLLQAVKRLQMEKLAVLLLIAGGNKYGINQETPYVSHLKREAVSLKDSVRFLPY  266

Query  63   IPSERAERVIPASRI--------EATKRAATIEATREPRTIVRGEGAGQAIETDVRETNI  114
            IP +   ++ PA+ +        EA      +EA      +V     G      +RE  I
Sbjct  267  IPHDEIHQLYPAADLFLMPSIGFEAFG-LVNLEAMSSCLPVVASRNGG------IRE--I  317

Query  115  TRHESSGMENPRYEDLPMLSVV  136
             RHE  G+  P  E  P++  V
Sbjct  318  IRHEKEGLLVPIGEPEPIVQAV  339


>emb|CEL57395.1| hypothetical protein RSOLAG1IB_02134 [Rhizoctonia solani AG-1 
IB]
Length=705

 Score = 41.2 bits (95),  Expect = 3.5, Method: Compositional matrix adjust.
 Identities = 21/41 (51%), Positives = 27/41 (66%), Gaps = 1/41 (2%)

Query  6   PTRESLQMLKRVSLQKLCKSLFIKGANKKSVEIIEVILAHY  46
           PTR  L+ LKR +LQ  CK L IK AN KS  +I+++L  Y
Sbjct  4   PTRVELENLKRAALQAKCKELGIK-ANSKSEHLIDMVLERY  43


>ref|WP_005581872.1| Group 1 glycosyl transferase [Clostridium ultunense]
 emb|CCQ92380.1| Group 1 glycosyl transferase [Clostridium ultunense Esp]
Length=381

 Score = 40.8 bits (94),  Expect = 4.2, Method: Compositional matrix adjust.
 Identities = 37/142 (26%), Positives = 63/142 (44%), Gaps = 18/142 (13%)

Query  3    QKGPTRESLQMLKRVSLQKLCKSLFIKGANKKSVEIIEVILAHYAQKDIEVEEAKQIAPV  62
            QKG     LQ +KR+ ++KL   L I G NK  +      ++H  ++ + ++++ +  P 
Sbjct  208  QKG-VHLLLQAVKRLQMEKLAFLLLIAGGNKYGINRETPYVSHLKREAVSLKDSVRFLPY  266

Query  63   IPSERAERVIPASRI--------EATKRAATIEATREPRTIVRGEGAGQAIETDVRETNI  114
            IP +   ++ PA+ +        EA      +EA      +V     G      +RE  I
Sbjct  267  IPHDEIHQLYPAADLFLMPSIGFEAFG-LVNLEAMSSCLPVVASRNGG------IRE--I  317

Query  115  TRHESSGMENPRYEDLPMLSVV  136
             RHE  G+  P  E  P++  V
Sbjct  318  IRHEKEGLLVPIGEPEPIVQAV  339


>ref|WP_032405444.1| hypothetical protein [Rhodococcus fascians]
Length=251

 Score = 39.7 bits (91),  Expect = 6.4, Method: Compositional matrix adjust.
 Identities = 52/207 (25%), Positives = 83/207 (40%), Gaps = 42/207 (20%)

Query  193  IYRDPSTPEQNFLAGPSMLGAAPIQLLTEQLSVNKNVLTVMESIMPLRGNKVKIRSHPFV  252
            + R P   E N   G +  G + +Q +   +  +  VLTV               S P +
Sbjct  59   LRRRPELAESNLFCG-NAFGGSVVQAMLPAVGPDAAVLTV---------------SAPTI  102

Query  253  AASMASMSKRTRCLGSQLLAGRIRKEMNDRD--QRLHPRASLSVLGKRQRLNDAKSLVFR  310
            A   AS+++R   +       RI   +   D   R H   S SV G   R++D      R
Sbjct  103  AD--ASLAQRLGEVAELARHRRIDDALRTLDFYVRSHFVRSPSVEGAATRIDDIDDACRR  160

Query  311  VSKRLSSLTAVELSS----HIPKTLSVLGKRAR---EPTFSIPEYEFADEFSFPLPPVLR  363
            +   +S LT+++L S    H    L+++G+ +R     T  +PE+               
Sbjct  161  IEMGMSMLTSIDLDSVVADHRGPMLAIVGQESRLVTTATVRVPEHGV-------------  207

Query  364  LATPP--IMDPHKDPWPSVGPLLSEPP  388
            +A  P   M PH+D WP+V   L+  P
Sbjct  208  IADIPRSGMRPHRDNWPAVARALAAHP  234


Lambda      K        H        a         alpha
   0.318    0.133    0.394    0.792     4.96 

Gapped
Lambda      K        H        a         alpha    sigma
   0.267   0.0410    0.140     1.90     42.6     43.6 

Effective search space used: 6683879375052


  Database: nr
    Posted date:  Sep 23, 2015 12:05 AM
  Number of letters in database: 26,053,659,533
  Number of sequences in database:  71,551,133


Matrix: BLOSUM62
Gap Penalties: Existence: 11, Extension: 1
Neighboring words threshold: 11
Window for multiple hits: 40
```
